# Supplementary material for: Domain-PFP allows protein function prediction using function-aware domain embedding representations
Source: Commun Biol. 2023 Oct 31;6:1103. doi: 10.1038/s42003-023-05476-9 (PMC10618451; doi:10.1038/s42003-023-05476-9)
Supplement: Supplementary file 2 — Supplementary Information [file 42003_2023_5476_MOESM2_ESM.pdf]

Supplementary Information for

Domain-PFP allows Protein Function Prediction Using Function-Aware Domain Embedding Representations

Nabil Ibtehaz[1], Yuki Kagaya[2], & Daisuke Kihara[1,2,*]

[1] Department of Computer Science, Purdue University, West Lafayette, Indiana, 47907, USA
[2] Department of Biological Sciences, Purdue University, West Lafayette, Indiana, 47907, USA
* Corresponding author. E-mail: dkihara@purdue.edu

Supplementary Table 1. Training Information of the 12 PLMs in PROBE benchmark.

Model	Embedding Size	Number of Parameters	Training Data	Unsupervised tasks used for training	Ref.
**ProtVec**	100	-	Swiss-Prot	word2vec	Asgari & Mofrad, 2015
**LearnedVec**	128	-	524,529 UniProt sequences	doc2vec	Yang et al., 2018
**UniRep**	5700	18.2M	UniRef50	masked language modelling	Alley et al., 2019
**SeqVec**	1024	93M	UniRef50	predicting the next amino acid	Heinzinger et al., 2019
**MSA-Transformer**	768	100M	UniRef50	masked MSA modelling	R. M. Rao et al., 2021
**CPCProt**	512	71M	Pfam	mutual information estimation	Choy et al., 2018
**TAPE-BERT-PFAM**	768	38M	Pfam	masked language modelling	R. Rao et al., 2019
**ProtBERT-BFD**	1024	420M	BFD	masked language modelling	Elnaggar et al., 2021
**ESM-1b**	1280	650M	UniRef50	masked language modelling	Rives et al., 2021
**ProtXLNet**	1024	409M	UniRef100	masked language modelling	Elnaggar et al., 2021
**ProtALBERT**	4096	224M	UniRef100	masked language modelling	Elnaggar et al., 2021
**ProtT5-XL**	1024	3B	UniRef50	masked language modelling	Elnaggar et al., 2021

References of the protein language models

Alley, E. C., Khimulya, G., Biswas, S., AlQuraishi, M., & Church, G. M. (2019). Unified rational protein engineering with sequence-based deep representation learning. *Nature Methods*, *16*(12), 1315–1322. https://doi.org/10.1038/s41592-019-0598-1

Asgari, E., & Mofrad, M. R. K. (2015). Continuous Distributed Representation of Biological Sequences for Deep Proteomics and Genomics. *PLOS ONE*, *10*(11), e0141287. https://doi.org/10.1371/journal.pone.0141287

Choy, C. T., Wong, C. H., & Chan, S. L. (2018). Infer related genes from large scale gene expression dataset with embedding. *BioRxiv*, 362848. https://doi.org/10.1101/362848

Elnaggar, A., Heinzinger, M., Dallago, C., Rehawi, G., Wang, Y., Jones, L., Gibbs, T., Feher, T., Angerer, C., Steinegger, M., Bhowmik, D., & Rost, B. (2021). ProtTrans: Towards Cracking the Language of Lifes Code Through Self-Supervised Deep Learning and High Performance Computing. *IEEE Transactions on Pattern Analysis and Machine Intelligence*, 1–1. https://doi.org/10.1109/TPAMI.2021.3095381

Heinzinger, M., Elnaggar, A., Wang, Y., Dallago, C., Nechaev, D., Matthes, F., & Rost, B. (2019). Modeling aspects of the language of life through transfer-learning protein sequences. *BMC Bioinformatics*, *20*(1), 723. https://doi.org/10.1186/s12859-019-3220-8

Rao, R., Bhattacharya, N., Thomas, N., Duan, Y., Chen, X., Canny, J., Abbeel, P., & Song, Y. S. (2019). Evaluating Protein Transfer Learning with TAPE. *Advances in Neural Information Processing Systems*, *32*, 9689–9701. https://pubmed.ncbi.nlm.nih.gov/33390682

Rao, R. M., Liu, J., Verkuil, R., Meier, J., Canny, J., Abbeel, P., Sercu, T., & Rives, A. (2021). MSA Transformer. In M. Meila & T. Zhang (Eds.), *Proceedings of the 38th International Conference on Machine Learning* (Vol. 139, pp. 8844–8856). PMLR. https://proceedings.mlr.press/v139/rao21a.html

Rives, A., Meier, J., Sercu, T., Goyal, S., Lin, Z., Liu, J., Guo, D., Ott, M., Zitnick, C. L., Ma, J., & Fergus, R. (2021). Biological structure and function emerge from scaling unsupervised learning to 250 million protein sequences. *Proceedings of the National Academy of Sciences*, *118*(15). https://doi.org/10.1073/pnas.2016239118

Yang, K. K., Wu, Z., Bedbrook, C. N., & Arnold, F. H. (2018). Learned protein embeddings for machine learning. *Bioinformatics*, *34*(15), 2642–2648. https://doi.org/10.1093/bioinformatics/bty178

Supplementary Table 2. Comparison with 12 PLMs in PROBE benchmark.

Model	MF	BP	CC	Avg.
**ProtVec**	0.64	0.36	0.38	0.46
**Learned-Vec**	0.68	0.39	0.41	0.49
**UniRep**	0.82	0.48	0.53	0.61
**SeqVec**	0.89	0.60	0.61	0.70
**MSA-Transformer**	0.67	0.47	0.50	0.55
**CPCProt**	0.65	0.40	0.44	0.50
**TAPE-BERT-PFAM**	0.85	0.54	0.58	0.65
**ProtBERT-BFD**	0.85	0.61	0.62	0.69
**ESM-1b**	0.83	0.53	0.61	0.66
**ProtXLNet**	0.82	0.50	0.59	0.63
**ProtALBERT**	0.89	0.63	0.64	0.72
**ProtT5-XL**	0.90	0.66	0.68	0.75
**DomainGO-Prob**	**0.92**	**0.72**	**0.74**	**0.79**

The value of F1 score is provided for each of the sub-ontologies along with their average value. The scores are collected from Table 2 of Unsal et al., 2022.

Supplementary Table 3. Overview of NetGO2.0 Benchmark Dataset.

Ontology	Number of GO Terms	Number of Training Proteins	Number of Validation Proteins	Number of Test Proteins
**MF**	6,854	62,646	1,128	505
**BP**	21,814	89,828	1,124	491
**CC**	2,880	81,377	1,359	268

The NetGO2.0 benchmark adopts a timeline-based approach similar to CAFA. Experimentally annotated proteins were obtained from GOA and UniProtKB, and were split into training, validation, and test sets as follows:

1. Training proteins: All proteins annotated on or before December 2018.
2. Validation proteins: Proteins annotated from January 2019 to January 2020.
3. Testing proteins: Proteins annotated between February 2020 and October 2020.

Only GO terms supported by evidence codes EXP, IDA, IPI, IMP, IGI, IEP, TAS, and IC were considered, and the test set comprised proteins from the 17 species of the CAFA4 challenge. In accordance with CAFA guidelines, proteins annotated with only the GO term "protein binding" (GO:0005515) were excluded.

The NetGO2.0 benchmark dataset used in our work is using the same files that are provided in the google drive url (https://drive.google.com/drive/folders/1wSS-R335UcNMToMskx3dE4XcTaLvCAOc).
But we used the NetGO2.0 dataset that was split into training, validation and test sets in the DeepGOZero paper by the Hoendorf lab, which they made available at (https://deepgo.cbrc.kaust.edu.sa/data/deepgozero/data-netgo.tar.gz). They made this split by following exactly the same procedure described in the NetGO2.0 paper and benchmarked recent methods on that, which enabled us to compare the performance of Domain-PFP against them.

Supplementary Figure 1. Selection of K in the KNN model of Domain-PFP.

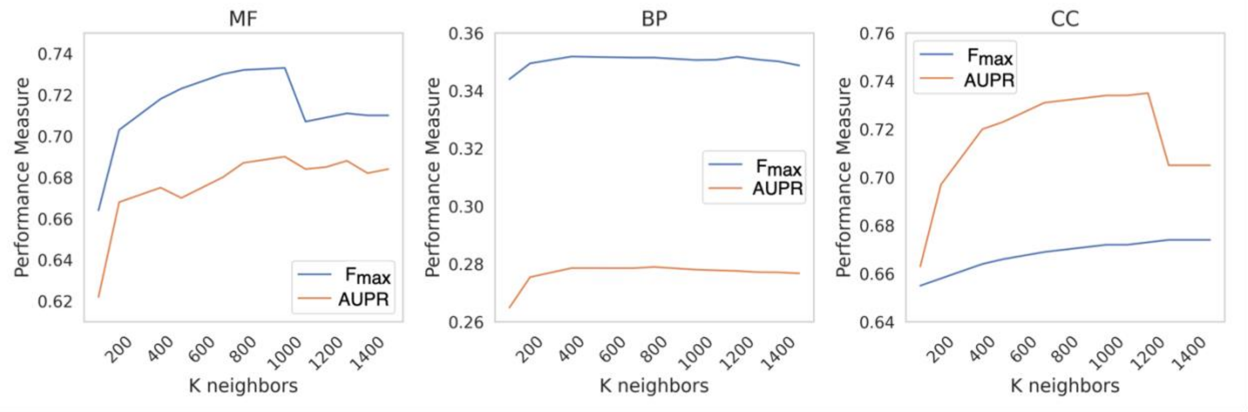

The value of of K for the KNN model was selected by observing the performance for different choices of K on the validation split of the NetGO benchmark dataset. The values of K were selected in the range from 100 to 1500 at an interval of 100 and for each value of K the F_{max} and AUPR scores on the validation data were computed.

We sought to select a value of K that manages to maximize both F_{max} and AUPR scores with more importance put on F_{max}. For MF, it was observed that the highest value of F_{max} was obtained with K=1000, after which there was a sharp decline of both F_{max} and AUPR. Thus, we selected K=1000 for MF. For BP, the change in performance for the different values of K was almost minute. We chose K= 800 because the performance was marginally better than the other values for K. For CC, similar to MF, the highest value of F_{max} was observed at K=1200, which dropped sharply with larger K. Although AUPR kept on improving even after K=1200, we choose K=1200 because the increment in AUPR was minor in comparison with the sharp drop in F_{max}.

Supplementary Table 4. Results on the Original NetGO Benchmark

Method	F_{max}			AUPR		
	**MF**	**BP**	**CC**	**MF**	**BP**	**CC**
Naive	0.416	0.256	0.542	0.276	0.118	0.464
BLAST-KNN	0.632	0.312	0.566	0.542	0.132	0.405
LR-3mer	0.427	0.258	0.552	0.317	0.125	0.478
LR-InterPro	0.651	0.325	0.641	0.623	0.166	0.587
Net-KNN	0.519	0.325	0.596	0.416	0.192	0.528
RNN	0.524	0.265	0.574	0.424	0.124	0.477
LR-Text	0.464	0.248	0.479	0.353	0.154	0.403
DeepGOPlus	0.620	0.305	0.620	0.521	0.115	0.493
GOLabeler	0.667	0.326	0.631	0.647	0.193	0.557
NetGO	0.674	0.362	0.646	0.653	0.239	0.583
NetGO 2.0	0.666	0.366	0.663	0.655	0.269	0.593
Domain-PFP	0.668	0.382	0.672	0.666	0.322	0.689
Domain-PFP + BLAST	0.668	0.407	0.677	0.688	0.344	**0.709**
Domain-PFP + PPI	0.651	0.409	0.667	0.672	0.353	0.664
Domain-PFP + BLAST + PPI	**0.675**	**0.425**	**0.678**	**0.708**	**0.365**	0.676

We evaluated Domain-PFP on the same train-validation-test split used in the original NetGO benchmark. Results of Naive to NetGO2.0 were taken from the NetGO2.0 paper. The top three scores for each of the metrics are highlighted by bold font, double underline, and single underline, respectively.

Supplementary Table 5. Comparison with structure-based function prediction methods

Method	F_{max}			AUPR		
	**MF**	**BP**	**CC**	**MF**	**BP**	**CC**
Naive	0.156	0.244	0.318	0.075	0.131	0.158
BLAST	0.498	0.400	0.398	0.120	0.120	0.163
DeepGO	0.359	0.295	0.420	0.368	0.210	0.302
DeepFRI	0.542	0.425	0.424	0.313	0.159	0.193
GAT-GO	0.633	0.492	0.547	0.660	0.381	0.479
Domain-PFP	0.662	0.519	0.532	0.653	0.503	0.510
Domain-PFP + BLAST	**0.746**	**0.611**	**0.605**	**0.754**	**0.606**	**0.612**

We retrained Domain-PFP on the same dataset used in the DeepFRI (Gligorijević et al., 2021) and GAT-GO (Lai & Xu, 2022) papers. Results of Naive to GAT-GO are taken from the GAT-GO paper.
The top three scores for each of the metrics are highlighted by bold font, double underline, and single underline, respectively.

Supplementary Table 6. Overview of CAFA3 Benchmark Dataset.

Ontology	Number of GO Terms	Number of Training Proteins	Number of Test Proteins
**MF**	677	36,110	1,137
**BP**	3,992	53,500	2,392
**CC**	551	50,596	1,265

The CAFA3 challenge organizers developed a training dataset with 66,841 protein sequences and their experimentally annotated functions (EXP, IDA, IPI, IMP, IGI, IEP, TAS, IC evidence codes). The training dataset was released in September 2016, which we used to train our model. A test dataset was provided to evaluate the models, which contains 'no-knowledge' proteins that were annotated experimentally between September 2016 and November 2017.
