# Supplementary material for: Domain-PFP allows protein function prediction using function-aware domain embedding representations
Source: Commun Biol. 2023 Oct 31;6:1103. doi: 10.1038/s42003-023-05476-9 (PMC10618451; doi:10.1038/s42003-023-05476-9)
Supplement: Supplementary file 3 — Reporting-Summary [file 42003_2023_5476_MOESM3_ESM.pdf]

nature research

Corresponding author(s): Daisuke Kihara

Last updated by author(s): 10/13/2023

Reporting Summary

Nature Research wishes to improve the reproducibility of the work that we publish. This form provides structure for consistency and transparency in reporting. For further information on Nature Research policies, see our Editorial Policies and the Editorial Policy Checklist.

Statistics

For all statistical analyses, confirm that the following items are present in the figure legend, table legend, main text, or Methods section.

n/a	Confirmed	
☐	☒	The exact sample size (n) for each experimental group/condition, given as a discrete number and unit of measurement
☐	☒	A statement on whether measurements were taken from distinct samples or whether the same sample was measured repeatedly
☒	☐	The statistical test(s) used AND whether they are one- or two-sided<br>*Only common tests should be described solely by name; describe more complex techniques in the Methods section.*
☒	☐	A description of all covariates tested
☒	☐	A description of any assumptions or corrections, such as tests of normality and adjustment for multiple comparisons
☒	☐	A full description of the statistical parameters including central tendency (e.g. means) or other basic estimates (e.g. regression coefficient) AND variation (e.g. standard deviation) or associated estimates of uncertainty (e.g. confidence intervals)
☒	☐	For null hypothesis testing, the test statistic (e.g. F, t, r) with confidence intervals, effect sizes, degrees of freedom and P value noted<br>*Give P values as exact values whenever suitable.*
☒	☐	For Bayesian analysis, information on the choice of priors and Markov chain Monte Carlo settings
☒	☐	For hierarchical and complex designs, identification of the appropriate level for tests and full reporting of outcomes
☒	☐	Estimates of effect sizes (e.g. Cohen's d, Pearson's r), indicating how they were calculated

Our web collection on statistics for biologists contains articles on many of the points above.

Software and code

Policy information about availability of computer code

Data collection	n/a
Data analysis	The program is freely available for academic use via Github, https://github.com/kiharalab/Domain-PFP and Google Colab Web server: https://bit.ly/domain-pfp-colab

For manuscripts utilizing custom algorithms or software that are central to the research but not yet described in published literature, software must be made available to editors and reviewers. We strongly encourage code deposition in a community repository (e.g. GitHub). See the Nature Research guidelines for submitting code & software for further information.

Data

Policy information about availability of data

All manuscripts must include a data availability statement. This statement should provide the following information, where applicable:

- Accession codes, unique identifiers, or web links for publicly available datasets
- A list of figures that have associated raw data
- A description of any restrictions on data availability

All the datasets used in this study are publicly available:
1. Swiss-Prot (https://www.uniprot.org/uniprotkb?query=reviewed:true)
2. InterPro2GO (https://www.ebi.ac.uk/GOA/InterPro2GO)
3. PROBE benchmark dataset (https://github.com/kansil/PROBE)
4. NetGO2.0 dataset (https://drive.google.com/drive/folders/1wSS-R335UcNMToMskx3dE4XcTaLvCAOc), parsed by the Hoendorf lab who used the dataset in their work of DeepGOZero. The parsed dataset we used is available at (https://deepgo.cbrc.kaust.edu.sa/data/deepgozero/data-netgo.tar.gz)
5. CAFA3 benchmark dataset (https://biofunctionprediction.org/cafa/)

6. the snapshot of the code at the time of acceptance https://doi.org/10.5281/zenodo.8436582
7. embeddings https://doi.org/10.6084/m9.figshare.24302845

Field-specific reporting

Please select the one below that is the best fit for your research. If you are not sure, read the appropriate sections before making your selection.

☒ Life sciences ☐ Behavioural & social sciences ☐ Ecological, evolutionary & environmental sciences

For a reference copy of the document with all sections, see nature.com/documents/nr-reporting-summary-flat.pdf

Life sciences study design

All studies must disclose on these points even when the disclosure is negative.

Sample size	Our model was initially trained on proteins sequences from the Swiss-Prot database, a total of 531,599 proteins were used in our initial experiments. For evaluations on the PROBE benchmark, we removed 19,996 human proteins from the initial dataset. Later, we evaluated our method on NetGO2 and CAFA3 benchmarks the sample sizes of which are presented in SM Table 3 and SM Table 4, respectively.
Data exclusions	The proteins that did not have any InterPro domains were excluded from the training data. However, proteins with missing or no InterPro domains were not removed from the test data.
Replication	Deep learning models were trained in non-deterministic manner, therefore the results are not fully replicable. However, the model weights are provided using which the results presented in the paper can be reproduced.
Randomization	The assignment of the samples to the different groups were not performed randomly, rather they were done following the standard timeline based cut-off for protein function prediction. SM Table 3 and SM Table 4 describe these assignments.
Blinding	The group allocations were performed in previous benchmarking works.

Reporting for specific materials, systems and methods

We require information from authors about some types of materials, experimental systems and methods used in many studies. Here, indicate whether each material, system or method listed is relevant to your study. If you are not sure if a list item applies to your research, read the appropriate section before selecting a response.

Materials & experimental systems

n/a	Involved in the study	
☒	☐	Antibodies
☒	☐	Eukaryotic cell lines
☒	☐	Palaeontology and archaeology
☒	☐	Animals and other organisms
☒	☐	Human research participants
☒	☐	Clinical data
☒	☐	Dual use research of concern

Methods

n/a	Involved in the study	
☒	☐	ChIP-seq
☒	☐	Flow cytometry
☒	☐	MRI-based neuroimaging
